# Migrant physicians’ conceptions of working in rural and remote areas in Sweden: A qualitative study

**DOI:** 10.1371/journal.pone.0210598

**Published:** 2019-01-14

**Authors:** Linda Sturesson, Magnus Öhlander, Gunnar Nilsson, Terese Stenfors

**Affiliations:** 1 Department of Learning, Informatics, Management and Ethics, Karolinska Institutet, Stockholm, Sweden; 2 Department of Ethnology, History of Religions and Gender Studies, Stockholm University, Stockholm, Sweden; 3 Department of Neurobiology, Care Sciences and Society, Karolinska Institutet, Stockholm, Sweden; Nord University, NORWAY

## Abstract

**Objective:**

To explore migrant physicians’ conceptions about working in rural and remote areas in Sweden to understand what influences their motivation to work in these areas.

**Method and material:**

The study employed a qualitative approach with semi-structured interviews with 24 migrant physicians. Transcripts were thematically analysed.

**Results:**

Conceptions were identified about foremost work content and tasks, and about living in rural and remote areas. Work content and tasks related to the health care systems, type of health care facility, duties, specialty, resources, patient population, colleagues, and professional development. Conceptions about living concerned geographical characteristics, people living in rural and remote areas, opportunities for travelling, family, leisure activities, social life, and language skills. Conceptions seemed to be influenced by individual, professional and societal aspects from both previous countries and Sweden. Conceptions and biographical aspects both appeared to affect motivation.

**Discussion:**

Motivation regarding working in rural and remote areas appeared to be influenced by conceptions of these areas. A specific type of place could be understood as being able to provide (or not) the external conditions needed for fulfilling needs and reaching goals, whether professional or personal, and as a tool for reaching or facilitating the achievement of these. Conceptions of an area can hence affect motivation and choices for where to work and live. However, biographical aspects also impact motivation. Our results indicate that positive rural experience in the recipient country might be a predictor for motivation.

**Conclusion:**

Professional and personal life and are intertwined. Conceptions about an area influence willingness to work there. Willingness is also affected by, and intertwined with, other aspects such as previous experiences, age, marital status and family circumstances.

## Introduction

The absence of physicians in rural and remote areas is a global challenge [1–9]. When care cannot be provided to the extent needed in these areas, public health is endangered and social is reinforced. Different initiatives have been undertaken to address the shortage of physicians in rural areas, including rural practice during medical education [8], financial incentives [8] or supporting physician´s professional development in rural and remote areas via interventions [10]. Another strategy is to establish cooperation and networks among universities, governments and rural areas [11]. However, a shortage of physicians in rural areas still remains. The strongest predictor for working as a physician in a rural area is having been raised in such an area [7, 8]. However, for International Medical Graduates (IMGs), this correlation is not significant, suggesting that other aspects affect their choice of where to work [8]. Some challenges of working as a physician in rural and remote areas have been highlighted, such as longer distances to larger cities, adverse location characteristics and fewer opportunities for professional development and limited opportunities for physicians’ families [1, 8, 9]. Regarding IMGs specifically, factors that have been identified affecting their attitudes towards working in rural areas include aspects related to their employment and career plans, but also to the families in terms of spousal employment and schooling [5]. Furthermore, access to cultural or religious foods, goods or services has been identified as an influential factor [5]. Internationally educated health professionals tend to live and work in urban areas [5, 9, 12], as migrants in general do [13] even though the notion of `rural´ often carries idyllic and positive connotations, at least in the west of Europe [14–16].

International migration is a well-explored research field. Aspects of migration to rural areas have been explored using, for example, a life course perspective [15] and the complex concept of rural has been analysed from the perspective of social representation [14]. However, studies with these types of theoretical underpinnings seldom seem to focus on a particular profession or on international migrants and their conceptions about of practising in rural areas of their recipient country.

Moving from one place to another is a major life event (Fielding, cited in [15]) and everyday life in the new location is a major concern (cf. [15]). A specific type of area can be thought of as being able to provide (or not) the external conditions necessary to fulfil needs and reach goals, whether professional or private (cf. [17–19]). Thoughts or conceptions about an area can hence affect internal motivation and choices regarding where to work geographically (cf. [20]).

A shortage of physicians in rural and remote areas is also a problem in Sweden [21]. Hence, this study aimed to explore non-established physicians’ conceptions about practising in rural and remote areas in Sweden since this, to our knowledge, is unexplored. The analytical basis for the study was that migrant physicians’ (MPs) understanding of work-life in rural and remote areas influences their choices regarding geographical location for where to work and live. This study focused specifically on non-established MPs in light of the scarcity of empirical studies which have explored their conceptions, especially in Sweden. Working as a physician in rural and remote areas can shorten a MP’s establishment phase, since doing this generally decreases the time to acquire a Swedish medical license. Yet, early establishment in the labour market is important for society, integration and well-being, as well as for maintaining professional knowledge and skills. Once conceptions of working as a physician in rural and remote areas are identified, they may be addressed by employers and others via interventions to recruit and retain physicians. A qualitative approach was found to be appropriate for revealing unexpected or unexplored conceptions, for visualising their variety, and for identify patterns [22], in experiences and conceptions that could influence motivation.

In qualitative research, contextualisation is important for trustworthiness [22]. Hence, the notion of rural will be discussed, as will the characteristics of the MPs included in this study. The paper presents the variety of conceptions among MPs about working in rural and remote areas and address how these conceptions could contribute to the willingness or reluctance to work in these areas. Furthermore, the paper analyses a few individual cases in more detail to explore how a person´s previous life experience, age, marital status and family can potentially explain their choices regarding working and living in rural areas. Doing so will yield further insights into the interplay between, on the one hand, individual MPs’ life stories and living circumstances, and on the other, conceptions about rural areas found in the material. Overall, the intention of this work, however, is to reduce the scarcity of physicians in rural and remote areas and facilitate MPs’ establishment in the labour market.

## Material and methods

A qualitative approach with semi-structured interviews was used in this study, and thematic analysis was employed to interpret the data. The guidelines for qualitative research as described in Tong et al. [23] were followed. Ethical approval was received from the Regional Ethical Board in Stockholm, Sweden (2017/1717–31/5).

### Theoretical underpinning and definitions

The results of this study describe conceptions about working in rural and remote areas. In this study, we define *conceptions* as thoughts, ideas and understandings. Having conceptions about a phenomenon does not necessarily require personal experience with it [18, 19]. An assumption of this study was that conceptions play a role in making choices regarding one’s future career (cf. [17]). The study was influenced by sociologist Stuart Hall who argued that phenomena, as well as things and people, are loaded with meanings by individuals, and that ‘we use the principles of similarity and differences to establish relationships between concepts or to distinguish them from one another’ [18, 19]. Hall suggested that concepts are formed for both concrete and abstract things as well as for people and places regardless of one’s level of experience with them, since meaning is always produced and exchanged in ‘different sites and circulated through several different processes or practices (the cultural circuit)’ (e.g. in media and social interactions) [18, 19]. In this article the terms biographical aspects are used to represent MPs’ life stories including previous experiences and their living circumstances as well as their age, marital status and family circumstances.

#### Setting—What is rural and what is not?

Including a rural perspective in research requires a discussion about the term rural. What constitutes a rural area is not the same across the world nor has it been through time [2, 24]. Thus, it is a contextual term. To define rural, different criteria are used, such as population size and geographic distance, which, in combination, represent population density [2]. Criteria might also include distance to urban areas of different sizes, types of services available, and the size and breadth of the local labour market [25]. In Sweden, *urban* is defined as an area with at least 200 inhabitants in which the distance between houses is less than 200 metres [26], but the criteria used for defining rural, remote and countryside vary among authorities [27]. However, most municipalities in the northern health care region of Sweden are defined as countryside or rural [26]. The geographic area comprising the northern health care region is approximately one half the size of Sweden, and yet the population of the region is just 9% of the total Swedish population [28]. In this study, smaller urban areas with less than 20,000 inhabitants are included in the conception of rural and remote.

As outlined above, rural can be defined by measurements, such as geographical proximity to urban areas. Rural can also be defined as a space or as a concept, including associated notions of rural (cf.[14]). For example, through the lens of social representation, ‘the rural consists of both abstract concepts and concreate images’ [14]. By using the perspective of social representation, a researcher can define rural based on individual´s talk about it [14].

### Participants

For this study, 24 MPs with a medical degree from outside the European Union/European Economic Area (EU/EEA) were interviewed. The participants were recruited via the complementary programme (CPP), which is one of three pathways for obtaining a Swedish medical license for physicians educated outside the EU/EEA. At the time of this study, the CPP was offered at three universities located in urban areas in Sweden. Before admission to the CPP, applicants receive a merit evaluation and are interviewed as a part of the selection process. Anyone admitted to the CPP must have formal qualifications in the Swedish language. Within two weeks from the CPP start, the study was presented to the 62 CPP participants with an invitation to participate in this study. A total of 24 physicians volunteered for the study. All participants provided formal written consent before participation.

### Data collection

During the data collection phase, interviews were conducted, interview transcripts were reviewed, and an interview guide was gradually refined. The interview questions focused on working and living experiences in previous countries and in Sweden, as well as future career plans, and conceptions about working as a physician in rural and remote areas in comparison to working in urban areas, both in Sweden and in previous countries. The interviews lasted 27–91 minutes and were conducted in Swedish. One half of the interviews lasted longer than 60 minutes. Of the interviews, 23 were conducted face-to-face at the participants’ universities, and one was conducted via Skype. The interviews were audio recorded and transcribed verbatim.

### Analysis

The analytical process for identifying conceptions followed the principles of thematic content analysis [29]. Focus was placed on the utterance level and on textual analysis, which is one part of sociological discourse analysis, together with contextual and sociological analysis [30]. Transcripts of the first 18 interviews were read through once to gain a first impression of the themes of conceptions that could be identified in the material. These transcripts were then re-read twice, and key words/codes related to the semantic content were noted on a coding sheet. In another section of the coding sheet, the researchers’ thoughts and interpretations (the latent manifest) of the semantic content in relationship to the entire interview were noted. Transcripts were then imported to the qualitative software programme NVivo 11. Based on the coding sheet, nodes and sub-nodes were created. In NVivo 11, the material was read through and coded into units of meaning. When nodes overlapped, they were merged. After reviewing and coding the first 18 interviews, the final six interviews were read through and coded with no new codes noted. These six interviews were also exported to NVivo 11 and again coded to the existing nodes. The coding was continuously reviewed by the last author to increase the trustworthiness of the results.

Finally, a thematic analysis [31] was conducted with the aim of exploring whether–and if so, which–conceptions influenced motivations, positively or negatively, to work in different areas, as well as to identify any patterns with respect to biographical aspects that could influence conceptions and, foremost, motivation. In this process, the transcripts were analysed as cases and read through once for familiarisation. Patterns and influential aspects were identified as the content was analysed and organised in spreadsheets. Even when an interviewee did not have explicit thoughts about working as a physician in rural or remote Sweden, they were still included in the dataset, since their experiences and thoughts in other matters expanded and deepened the understanding and interpretation of the material.

#### Participant´s characteristics

Table 1 shows some interviewees’ characteristics. Common among them was that MPs were not primarily workforce immigrants and had not yet worked as licensed physicians in Sweden; however, some had prior experience with Swedish health care before entering the CPP such as working as another type of health care professional (usually as an assistant nurse or assistant physician), practise, and visiting a doctor as a patient or relative.

**Table 1:** Interviewees characteristics (*N* = 24).

Male	14
Female	10
Age (average)	33
Number of countries (growing up)	11
Number of countries (medical education)	13
Number of years since medical school graduation (average)	8
Number of physicians with internship and/or working experience in areas with fewer than 10,000 inhabitants in a previous country	2
Number of physicians with internship and/or working experience in areas with fewer than 25,000 inhabitants in a previous country	1
Specialists or had begun specialist training in a previous country	9
Number of years in Sweden	2–10
Physicians with living experience in areas with fewer than 20,000 inhabitants in Sweden	10
Physicians with working or personal experience with health care in Sweden before entering the CPP	22

Experiences of living and working in rural and remote areas in previous countries were few, while the reasons given for living and working in these areas included political reasons, moving back home, and conditions in state-financed medical education. MPs worked in primary care, at a minor hospital or at a regional hospital, in most of the cases serving a larger area with many patients. The reasons given for leaving rural areas included heavy workloads, lack of professional development opportunities, missing family, believing there was more to life in another area, and migration due to war.

MPs had conceptions about physicians working in rural and remote areas as such in previous countries, and the work as physicians in these areas. In many of the MPs countries of origin, physicians were said to want to work in urban areas since larger hospitals were situated in these areas. Larger hospitals were associated with higher status, higher salaries, well-educated colleagues and better resources. Resources such as infrastructure and equipment were thought to be lacking in rural areas in comparison to urban areas. For some, this meant that working as a physician in rural areas mainly consisted of referring patients. For others, working in rural areas implied more responsibility due to the lack of resources and occasionally being the only resident physician. This was thought by some to increase work stress but also to lead to the development of experience. Being a general practitioner (GP) was, in some countries, not a specialty, and physicians working as GPs in some previous countries were seen as less skilled or less educated.

Reasons given for why MPs lived or had lived in rural or smaller urban areas in Sweden were both involuntary and voluntary. As refugees, some were, at first, relocated to a remote or rural area. Some MPs moved to be with spouses already living in rural areas, while others moved because it was easier to find accommodation. Reasons for leaving rural and remote areas for larger urban areas in Sweden included wanting to be closer to peers from previous countries, seeking increased opportunities for finding work, learning Swedish and participating in the CPP. Some were still living in rural or smaller urban areas during the CPP but were within a commuting distance.

#### Speaking about rural areas–conceptions of the setting

During the interviews, the complexity and relativity of the concepts or remote, rural and urban were revealed. Different references, comparisons and value-laden words were used to reach an understanding between the researcher and the MPs. Comparisons and differences between urban areas in previous countries and in Sweden were often used when conceptions of working and living in rural and remote areas were articulated. Measurements, such as population size, were common, and some mentioned the distance to certain cities. Mentioning a city as the second or third city in a country was sometimes used for explaining size. This reference was also used for comparisons between countries even if the size of the population differed. Value-laden words, such as ‘very small’, ‘small’, ‘large’ or ‘big’ were used. Conceptions about what was rural and remote were relative and contextual and could sometimes be related to feelings or available resources in the area (i.e. Internet and the ability to buy what was needed). One physician compared two urban areas with the same population size, one in Sweden and the other in a previous country, stating that there was a difference regarding quality of life, was thought as higher in Sweden even if of the population size was the same: ‘I'm sure that […], if I live in [previous country], in a city with [same population size as a city in Sweden] …I'm not going to have the same quality of the Internet and I cannot buy what I need’ (MP01). In comparison with larger cities in Sweden, infrastructure, such as trains and airports, were also mentioned as missing resources in one previous country, even though the city in question was the second largest in the country (MP12). Rural and remote areas existed in the MPs’ previous countries, but determining what was considered rural and remote was relative in terms of measurements. For example, the same population size could in one country be seen as the countryside while in another be defined as a city. Our study thus confirms the complexity of the terms *rural* and *remote*, implying that a shared definition would be difficult to reach as it is contextual [2, 26].

In Sweden, rural and remote areas were associated with different parts of the country, but were foremost associated with northern regions. MPs with experiences from rural or remote areas in Sweden referred to areas that differed widely in terms of population size from hundreds to tens of thousands. Living rural did not necessarily mean living remote. Living remote could mean living rural, or it could mean living in smaller urban areas. Some MPS associated rural and remote areas with northern Sweden.

## Results

A majority of the MPs had plans for their future careers as physicians in urbans areas of Sweden, although most felt that a temporary placement in a rural area would also be an option. Others had not yet determined their plans. Some mentioned smaller urban areas or the north of Sweden in general as examples of places where they never wanted to work or might consider working, while others said they would only move there ‘if they had to’.

### Conceptions of working as a physician in rural and remote areas in Sweden

We identified conceptions about *finding work* and *work content and tasks*. Thoughts about working as a physician in rural areas also gave rise to conceptions of *living in rural and remote areas*.

#### Conceptions of finding work

*Finding work* in rural and remote areas was thought of as easier. Once they complete the CPP, MPs must find an internship (AT) by themselves, a stipulation which also applies to physicians with a medical education completed in Sweden. Several interviewees believed it would take less time to find an AT in smaller urban areas, remote areas or northern parts of Sweden. In Sweden, it is common knowledge that it takes longer to find an AT in the largest urban areas. Hence, some suggested that conducting an AT in smaller urban, rural or remote areas would decrease the time to obtain a Swedish medical license, receive specialist training and become a specialist in Swedish terms. MPs who felt rushed to obtain a Swedish medical license and become specialists in Sweden seemed to be more motivated to live and work in rural and remote areas. This did not necessarily mean that they wanted to stay in such areas after completing AT. An absence of physicians in certain regions was, according to one MP, an argument against applying to work in these areas, since the MPs’ interpretation was that this absence meant that Swedish physicians did not want to work in these areas.

Lack of flexibility and a weaker job market in rural areas were also mentioned. These conditions create an inconvenience since it was thought that *few workplace options* existed in rural areas. Particularly if the MP had a family, there was a concern that the family would need to move if they wanted more career options: ‘I have to move the whole family there …and …my child has to change school, something like that …for me bigger cities are …definitely better’ (MP20). In this example, aspects of private life interacted with professional motivations. This MP had experience living in a rural area in the country of origin, but had moved to a large urban area to study. It was said that everyone in the country ‘wants to move to a bigger city’ because of increased opportunities for education, work options and development as a physician.

On the other hand, *salaries* were thought to be higher in rural and northern areas. MPs mentioned knowing (or knowing about) both Swedish-educated physicians and IMGs who had worked in the north for some years and then returned to urban areas wealthier than before. The opportunity to earn more money, however, did not seem to be the primary reason for applying to areas with a lack of physicians.

#### Conceptions of work content and tasks

Conceptions regarding *work content and tasks* were divided into overlapping subthemes: *health care system*, *type of health care facility*, *duties*, *specialty*, *resources*, *patient population*, *colleagues*, *development of knowledge and competence*, and *other health care professionals*.

The overarching *health care system*, with its regulations, and the ability to access digital medical records were understood as being the same all over Sweden. MPs associated health centres and smaller hospitals with rural and remote areas. Conceptions regarding the work at these facilities were often compared to working at larger (university) hospitals, which in Sweden, are located in larger urban areas. It was described as being quieter to work in a rural or remote area. Hospitals in rural areas were thought of as being small or as just being large health centres. Different types of health care facilities, their size and the types of areas in which they were located were hence associated with different duties, specialisations and opportunities for physicians. If working in a health centre, *duties* were mentioned as being the same or different depending on area type. Duties at a health centre, regardless of area type, were described by some as talking to patients, writing referrals and referring patients due to the assumption that limited resources for examining and treating patients existed in such centres. On the other hand, duties at a health centre located in a rural area were described by others as being varied and yielding the best opportunities to become excellent GPs in comparison to working as a GP in an urban area. Rural and remote areas were mainly associated with the *specialty* of general practice. Thus, some MPs thought that physicians with an interest in becoming a GP, or who were GPs, were most suited to work in rural and remote areas.

*Resources* that were described as being available regardless of area were Internet and air ambulances. Having the ability to call an air ambulance was mentioned by one MP as making them more confident to work as a physician in rural and remote areas in Sweden in comparison to working in such areas in a previous country. Working at a health centre in Sweden was said to have ‘loads of resources so you can do much more’ in comparison to a previous country (MP02). Resources that were believed by some MPs to potentially be lacking in rural and remote areas in Sweden were x-ray and magnetron x-ray equipment and computer tomography.

Working in a rural area, in general, was associated with having broad knowledge and the ability to handle various diseases since the *patient population* would vary greatly. This was described as affecting both work content and tasks, such that both would be more varied as well. One MP said, ‘you do a lot on spot’ (MP02). In contrast, other MPs thought that patients in rural areas could be described as homogeneous (e.g. consisting of older people) and therefore not providing variation. One MP suggested that, in rural areas, a physician has *fewer colleagues*. Having fewer colleagues was thought to mean working alone more often and not always having someone to turn to with questions about decision making. This was understood to involve increased responsibility and making the work more difficult: ‘It is more difficult to work in the countryside because you are more responsible there, because you are almost alone, there are not so many doctors. In big hospitals, there is always somebody you can ask…a consultant, other physicians, specialists’ (P06). Another MP brought up the responsibility of being the only physician with a patient suffering from a serious disease (P13). Working in a rural area, and hence having fewer colleagues, was also mentioned in terms of increased independence, experience and knowledge, all of which were described as necessary for becoming a good physician (P14). Physicians who wanted to develop broad knowledge and handle different diseases were seen as well suited for work in rural areas, since working in a larger (university) hospital was seen as intending to ‘go deep’, i.e., focusing on smaller topics (P03).

Working in rural or remote areas was mentioned as promoting the *development of knowledge and competence*, but only to a certain extent. Learning more was considered to be difficult due to too few colleagues, colleagues’ level of skill and the perceived simplicity of patient cases. Opportunities for obtaining some specialties, returning to an original specialty and developing excellence in specialties other than GP were thought to be lacking in rural and remote areas, but were considered possible at larger (university) hospitals, which, in Sweden, are located in larger urban areas. University hospitals were thought of as having resources for greater learning, such as professors, the latest methods and technology, and complicated patient cases, all of which were thought as missing in rural and remote areas.

#### Conceptions of living in rural and remote areas

Conceptions about working as a physician in rural areas also involved living in rural and remote areas. We found conceptions regarding *geographical characteristics*; *people living in rural and remote areas*; *opportunities for travelling*, *family*, *hobbies and leisure activities*, and *social life*; and *language skills*. These conceptions seemed to affect motivation for working in rural, remote and smaller urban areas.

*Geographical characteristics* included conceptions of rural and remote areas as being quiet and small. The climate, specifically in northern Sweden, was highlighted by several MPs, who thought of it as cold and dark. MPs mentioned them not being used to this type of weather, with one laughed and said, ‘what shall I do if it is very cold and I cannot do anything?’ (i.e., during free time) (MP 23).

Living in a rural and remote area was, in general, described as expensive. Living remotely was also understood as complicating travel, increasing both travel time and costs:

‘If I want to go to [mentions another European country] it is much cheaper here [urban area where the physician now lives] … you need more time to travel from [names a small town in a remote area] to [the capital of Sweden] and then via airplane to another …’ (P16). Another MP added, ‘To me, it is important that it is close to an international airport’ (P20).

Thoughts about the climate, as well as the costs and complications of travel negatively affected MPs’ motivation to work in the northern parts of Sweden. MPs stressed that they wanted to visit family and friends living in other countries without having to travel extra within Sweden to reach an international airport.

Rural and remote areas were described by some as not providing *opportunities for the family*, such as finding a partner or having opportunities for children to study at university nearby. One MP explained the importance of living close to university: ‘If I live in [mentions an area with less than 20 000 inhabitants] that means they [the children] will leave me alone at home and go elsewhere … but if, for example, living in [mentions a larger urban area with university] they will study at the university in [the larger urban area] and then we have the opportunity to live close to each other … so I will not lose my children in the future … and that is important to us … I don’t know how it is for the Swedes, maybe it … they do not care if their children go to other countries … but for us … that's really important.’ (P16). Such thoughts decreased motivation to live in rural and remote areas. Some referred to their culture when speaking about the importance of living close to their children who in the future would be studying at universities.

On the other hand, living in rural and remote areas was believed to present *opportunities for hobbies and leisure activities* related to nature and the physical surroundings. However, living rural and remote or in a small urban area was understood as not offering possibilities for other hobbies and leisure activities, such as playing a certain sport, visiting theatres and exhibitions, or eating out with friends (due to too few restaurants in rural, remote or small urban areas). Conceptions regarding such opportunities for hobbies and leisure influenced motivations for living in rural, remote and smaller urban areas in either direction, positive or negative, depending on what the MPs liked to do in their spare time. The distance to urban areas with urban-related opportunities also affected their motivation (e.g. living at commuter distance positively influenced motivation).

*People living in rural and remote areas* were considered to be older. The number of people living in such areas was, of course, thought of as being few to the degree that some MPs highlighted the importance of seeing people and street activity in order to feel alive. Some understood rural and remote areas as consisting of mainly Swedes, and not of peers from their home country. Therefore, one MP expressed concerns about being the only foreigner, while another MP worried about experiencing loneliness. On the other hand, some MPs thought that being the only foreigner would give them opportunities to develop knowledge about Swedish culture. A number of MPs considered people in the countryside to be friendlier than people in urban areas and thus easier to get to know. It was also suggested that it would be easier to find time for socialising, for inviting people home for dinner, and for being invited to dinner. Some MPs considered *opportunities for a social life* to be lacking in rural or remote areas, in terms of both having friends around from their previous countries and as in having places to socialise with friends, such as at restaurants. Who the MPs conceptualised as living in rural and remote areas, combined with who they wanted to socialise with, peers or locals, also seemed to influence their motivation to live in such areas.

Conceptions related to *language skills* were also mentioned. Some MPs thought that it would be difficult to understand the local dialect. One MP expressed how unpleasant it would feel to be the only one with an accent, a belief which is related to the conception that the people living in rural and remote areas are foremost Swedes. One MP had already experienced insecurity about being the only one speaking ‘bad Swedish’ amongst fluent-speaking Swedes while living in a smaller urban area, which amongst other reasons influenced this MP towards wanting to work in a larger urban area (MP17). Another MP expected it to be hard to learn the language in a small town, but someone else thought that it would be easy since the area was thought of as consisting mostly of Swedes.

### Aspects influencing conceptions of and motivation to work as a physician in rural and remote areas in Sweden

We found that the conceptions seemed to influence the motivation to work in these areas in the future, either positively or negatively. To understand individual motivations for wanting to work in certain areas in more detail, we hence also analysed the material as cases as it appeared that biographical aspects had an impact on motivations for where the MPs wanted to work as well as on their conceptions. For example, one MP believing there were no opportunities for university study in rural and remote areas had a decreased motivation for working in this type of area since the MP had children. The MP emphasized that living near their children was important for the MP as this was a part of their culture. Except biographical aspects, we also found that conceptions about working and the motivation to work as a physician in rural or remote areas seemed to be influenced by professional and societal factors from both Sweden and previous countries. Below we will present four of our study participants in more depth, and after we will address some patterns found regarding biographical, professional and societal aspects from previous countries and Sweden that seemed to influence conceptions and motivations.

MP case number A is a woman born and raised in an urban area in her native country. She had studied to be a physician, had already worked for some years, and had begun specialisation training at a university hospital in a larger urban area. Her husband, who is from the same country as her, had received a job opportunity in Sweden, and thus she moved to Sweden with him. In Sweden, they settled in an urban area and have lived there ever since. They have one child, who is in school. She had no intention of leaving the urban area when the interview took place. She doubted the wisdom of moving to a rural area, referring to potential complications regarding work, family and simply being different. She could consider living in a rural area in Sweden for a shorter period, but only if it if it reduced the time to receive her Swedish medical license. However, once she would obtain her license she would return to the urban area, because she enjoys living in a city and has also had experiences living in a large city in her origin country. In that country, she mentioned that there are large differences between working in rural areas compared to working in urban areas. She mentioned that resources and infrastructure were lacking in rural areas, and that most physicians wanted to work at larger hospitals in urban areas since there they would have more opportunities for development, their work environment would be better, and their salaries would be higher.

MP case number B is a man born and raised in an urban area in which he also worked as a specialist for several years; however, he had received his medical education in another urban area. Besides being a physician, he was also a husband and father. He had left his country of origin due to political instability, as had many others from the same area; he had also faced a long and arduous journey before reaching Sweden as a refugee. He first had to live in a refugee camp located in an area with few inhabitants, after which he moved to a smaller urban area, both in the north of Sweden. He had then moved south to where he knew peers from his original country lived and hoped that by doing so he would have the opportunity to work as a physician again. Before entering the CPP, he had been taking an internship as an assistant nurse at a hospital. He associated rural areas with the north of Sweden and used the cold weather as an argument against living there. He also brought up the issue of living remotely, far from universities where his children would study in the future, and expressed his desire to remain close to his family. Being rural as well as remote would also complicate travels to other countries, as it would increase costs and the duration of travel. He worried about getting old and having moved so many times, and now he just wanted to be settled somewhere with his family. He explained that in his country of origin, working in urban areas was better since the salary was higher and money was needed since there was no social security system in place, as there is in Sweden. However, he viewed doing clinical work in a rural or urban area as being the same as long as it was preceded in a hospital.

MP case number C is a woman from an urban area. She had completed her medical education in an urban area and had begun specialist training when she met a man from Sweden and decided to migrate to the country to live with him. The man lived in an area considered to be rural, but still at a commuting distance to an urban area. Just after arriving to Sweden, she began language studies and found work in the health care sector. For her, it is important to live at least at commuting distance to an area with a variety of cultural activities. Since the area in which she lives is sparsely populated and she does not have children, her and her spouse had considered moving to increase their work opportunities in the future. However, they did not necessarily need to live in the largest urban areas in Sweden, and had therefore considered living and working in the north of Sweden so long as the chosen area had a hospital at which she could continue with her specialty training. In her country of origin, a physician who works in a health centre ‘in the middle of nowhere’ cannot do much and the physician is ‘someone with nothing […] here [in Sweden] at a health centre there are a lot of resources and you can do so much more ‘. She also mentioned that in her country of origin, it is harder to receive education at a distance, since there might be no internet, even though this situation is improving.

MP case number D is a man raised in an urban area that, in his country of origin, was amongst the largest. He had finished his medical education and had worked for some years at a hospital serving a larger region in the same country. Due to political reasons, he had migrated to Sweden. In Sweden, he was first sent to a refugee camp located in a rural area, after which he had moved to a small urban area, where he had made friends and found work. Thereafter, he had moved to a larger urban area to attend the CPP and has considered staying in the area, since he has relatives living nearby. However, he would also like to return to the smaller urban area, since he has friends there and he enjoys the local scenery. He remarked that in his country of origin, as well as in Sweden, people in the countryside are easier to befriend and hang out with–that people are friendlier. He emphasised that people in Sweden might be less stressed in rural areas, as such areas are much quieter. He also believed that working as a physician in a rural area would be quieter as well, since the patients would be fewer and the physician could hence devote more time to each of them.

MPs (the whole data set) described themselves as city people (e.g. born, raised and lived in larger urban areas) or as being accustomed to and liking the ‘small’; biographical aspects that in turn influenced their motivation to work and live in such areas in Sweden. MPs who had migrated to Sweden because of a Swedish spouse and who had positive living experiences in rural, remote or smaller urban areas in Sweden seemed positive about staying or returning to these areas after the CPP. Reasons given for such positivity included preferring quiet or small areas, having local friends, thinking people were friendlier than people living in larger urban areas, and enjoyment of the surrounding nature. Despite these reasons, the rural or remote areas had to have some opportunities of importance for themselves or their family in order to motivate them to relocate there.

MPs who had been raised and lived in cities with millions of inhabitants and then had had more or less involuntarily experiences in Sweden of living in rural, remote and small urban areas did not seem keen on returning to them. However, they did view it as a temporary possibility. MPs with living and health care experience in rural and remote areas in Sweden seemed more motivated to work in these areas. MPs without work experience in rural and remote areas, or MPs who only had experience working or living in larger urban areas in Sweden and in previous countries and who had worked as specialists in larger urban areas, seemed more motivated to pursue a career in a larger urban areas in Sweden.

Age, marital status and family status also seemed to affect motivation. Some MPs said that they were too old and had moved too many times already–now, they just wanted to settle down. This decreased their motivation to work in rural and remote areas. On the other hand, other MPs had moved so many times that doing it again would not make any difference, especially if they were young and without a family. For some MPs, having children were a major reason to remain where they were currently living, particularly if their children were in school or had friends. Another MP living in an urban area wanted to stay there for several other reasons, such as having relatives in the area, being familiar with the surroundings, and wanting to do research in the future.

MPs considered working in rural areas in Sweden to be easier than doing so in previous countries, which was described as stressful and limiting since many resources for taking some samples or carry out examinations were missing. At the same time, some described this as being beneficial for developing skills, knowledge, independence and self-esteem.

Some of the MPs were specialists or had begun specialist training, and as such they wanted to pursue these specialities; this in turn influenced their motivation to work in specific areas. In some countries, GP is not a specialty, which negatively influenced some MPs’ motivation to work in areas of Sweden primarily associated with GP, even though MPs emphasised the importance of GP.

In Sweden, conceptions and motivation amongst MPs were influenced by their own experiences as well as those of others, such as colleagues and health care staff whom the MPs had met in the Swedish health care sector before the CPP, other CPP participants, teachers and clinical supervisors (even though they had only been in the programme for a few weeks when interviewed) and friends (peers from previous countries as well as Swedes). Also, physicians in general seemed to influence conceptions and motivations. For example, physicians who gave a high ranking to a remote hospital in the national ranking system for internships positively influenced the motivations of MPs. In contrast, physicians’ choice to work predominantly in urban areas, which has consequently led to a shortage of physicians in rural areas, negatively influenced some MPs: If other physicians did not want to work in these areas, for whatever reasons, then why would the MPs? On a professional level, the absence of physicians in certain areas has influenced conceptions and motivations regarding where to work in the future. Living and working experiences in previous countries and in Sweden seemed to influence conceptions about living and working as a physician in rural and remote areas in Sweden. Such experiences did not necessarily need to be their own. We found that most of the MPs had little experience living and working in rural and remote areas; still, they had conceptions about what it would be like, and these conceptions in turn influenced their motivations.

## Discussion

This study aimed to explore MPs’ conceptions about working in rural areas in Sweden. The results can be used to contribute to the understanding of MPs’ choices of geographical location for work and settlement in Sweden. Previous research has shown that MPs face other challenges besides those encountered by Swedish-educated physicians. These include social, emotional and career challenges [5, 20], such as lacking social networks and support [9, 32], language barriers [9, 32], discrimination and cultural differences with respect to work [33–36]. The latter two challenges were not prominent themes in our study, which could be because they are not area-specific, have not yet been experienced by the MPs, or were sensitive topics for the MPs to talk about and therefore avoided (cf. [20, 37]. Our results revealed a variety of conceptions about working as a physician in rural areas which seemed to influence motivations about where to work in the future.

Conceptions about working in rural areas related to the themes of finding work, work content and tasks, and living in rural areas, and some common features were identified. Rural, remote and smaller urban areas were mainly associated with health centres, smaller hospitals and GP. Conceptions about working as a physician were in some cases indirectly connected to rural and remote locations, since larger (and university) hospitals in Sweden are located in larger urban areas, and these hospitals were thought by some as offering better opportunities for further development of knowledge and competence. Rural and remote areas were understood as having fewer resources for further learning (e.g., aspects regarding patient population and the level of complicated cases, the number of colleagues and their competence, and the proximity to research and advanced equipment). For some, working in rural and remote areas seemed to imply decreased opportunities for obtaining and developing excellence in specialties other than GP. Since some of the MPs had begun specialist training or were already specialists and therefore wanted to pursue their careers, this influenced their motivation to work in specific areas. Our interpretation is that opportunities for professional development and learning were important motivators for where to work in the future. Salary did not seem to be a key factor, which is in line with previous research that has suggested that other factors related to private life are of greater importance in the long run [8, 20].

Our interpretation is that conceptions about working in rural areas were for many MPs intertwined with living in the same area. Living in a rural area could be viewed as a possibility if the area was also believed to present opportunities regarding various aspects of private life. Conceptions about living in rural areas included the following themes: *geographical characteristics*; *the people living in rural and remote areas*; *opportunities for travelling*, *family*, *hobbies and leisure activities*, and *social life*; and *language skills*. Our results are in line with Parlier et al., who suggested that the quality of rural life is one dimension needed to solidify a ‘rural physician identity’ [8]. Areas that could provide opportunities for personal interests–for example, nature related–increased motivation to work in rural and remote areas. Living (and also working) in rural or smaller urban areas was a possibility for some if services such as universities, theatres, exhibitions and restaurants were located at a commuting distance. This is consistent with previous research suggesting that has suggested that other aspects besides just professional considerations matter when considering where to work, such as demographics and infrastructure [20], as well as opportunities for spouses and children [7, 8]. The conceptions revealed can be compared to research which has addressed two ways `rural´ living is represented. On the one hand, there is the image of the `rural´ as being boring and lacking facilities and services easily found in urban areas [14, 16]. On the other hand, the `rural´ can be seen more positively as a place with a slower pace, where everybody knows each other, and opportunities for interests related to nature and outdoor life are abundant [14, 16]. The different views about what rural living is like with may also be applied to our theme of *work content and tasks*; for example, some MPs highlighted that it is quieter to work in a rural area. The identities of MP´s might be grounded in life styles and cultural activities which they might believe to be difficult to obtain in rural areas; moreover, they might also consider it too difficult to develop their professional identity in rural areas, depending on their goals. Research has identified challenges experienced specifically by IMGs regarding private life and living and working in rural areas, such as opportunities for family, travelling and language barriers [5, 10]. Our results suggest that these types of challenges might be on the minds of the MPs when they are considering where to work in the future. In some cases, we found that concerns could be related to their non-local backgrounds–for example, not being able to socialise with peers from the same country, looking different or being the only one with an accent. On the other hand, being unique in this way could also be seen as an opportunity to learn about Swedish culture and develop language skills. Previous research has acknowledged that being part of the community is an important dimension of quality in rural life [8] and for highly skilled migrants in general [38]. Our results add to previous research which has suggested that being able to have a social life is important.

Internal motivations regarding working and living in rural and remote areas appeared to be influenced by conceptions about these areas. A specific type of place was understood as being able to provide (or not) the external conditions needed to fulfil needs and reach goals, whether professional or private (cf. [17]). An area can hence be seen as a tool (or a strategy) for reaching or facilitating the achievement of goals. Conceptions about an area can thus affect internal motivations and choices about where to live and work. Working and living in different areas seemed to be valued differently. Our results indicate that living and working in urban areas were seen as more beneficial than living and working in rural and remote areas, and our interpretation of this is that a hierarchical dimension based on geographical location might emerge.

Conceptions were influenced by both personal experiences and the experiences of others in previous countries as well as in Sweden. We found that conceptions were only one component influencing motivations about where to work. In some cases, we identified conceptions that were intertwined with biographical aspects, influencing motivations either positively or negatively. Hence, to understand why someone chooses to work in rural and remote areas, his or her biographical aspects must be explored (cf. [15]).

Rural experience has in previous research been identified as the strongest predictor for rural work for in-country educated physicians [7, 8]. Our results indicate that positive rural experiences in the recipient country might be a predictor for physicians educated abroad, and may positively influence motivations for living and working in rural and remote areas. MPs who had moved to a rural or remote location in Sweden where they had a spouse living seemed positive about working in these areas. Opportunities to become part of society were likely easier for them than they are for refugees placed in rural and remote refugee settlements, in which they have little contact with the surrounding communities or with the Swedish health care context. Our results thus support Parlier et al., who suggested that positive rural experiences are one aspect needed to develop a ‘rural physician identity’ [8], which might in turn increase the chance of remaining in a rural area. Our interpretation is that this observation might also apply to MPs as well as to physicians educated in Sweden. Other factors attributed to increasing the likelihood of remaining in a rural area are, for example, developing a sense of belonging and being integrated into the community, as well as educational opportunities for children; something that solidifies the rural physician identity (cf. [8]). We found that MPs who conceived of rural areas as having fewer opportunities for their children had a decreased motivation to work in such areas.

MPs with rural health care experiences in Sweden mentioned that MPs without these experiences tended to base their conceptions of the Swedish context on experiences and conditions in previous countries (e.g. concerning resources). It was also expressed that physicians who had worked in rural areas in previous countries might have been affected by such experiences, and that their attitudes towards wanting to work in rural areas in Sweden might be biased accordingly. As new experiences are added in life, life circumstances and conceptions changes, the willingness or reluctance to work in a specific area might not be a firm decision for all future (cf. [15]).

### Implications for practice

Our results indicate that MPs with a positive experience of rural and remote areas in Sweden might be motivated to work in these areas. To facilitate recruitment, it is recommended that employers establish contacts with bridging programmes, such as the CPP, to recruit MPs (cf. [8, 11]). Bridging programmes might include rural and remote practice, which is a strategy used in medical education in some parts of the world [8]. Such a strategy can be used for creating positive experiences, as we found that such experiences seemed to positively influence motivations to work in rural areas. Professional development programmes aimed at supporting IMGs have been developed, and these often address issues of language and professional development [9, 10, 32]. Educational interventions for IMGs often assume group homogeneity [9, 32], but they could instead be tailored to suit MPs at an individual level since they represent a heterogeneous group with different life stories and living circumstances. Interventions for IMGs specifically in rural and remote areas tend to focus more on professional life [10, 32] than on other aspects of life. Addressing the aspects that we identified and themed under the category of living might be difficult to resolve at the employment level, but it might be possible if the whole society is involved. For example, Wright et al. showed that the reasons provided for not wanting to stay in rural areas included ‘a limited social network’ [10]. Being integrated into a social context might therefore be important, but it would be difficult to achieve without cooperation from the surrounding community (cf. [38]). It is recommended that aspects other than just professional concerns could be included in further developments.

### Limitations and strengths

This study was based on a heterogeneous group of 24 MPs from 11 different countries. As such, it is difficult to identify patterns regarding *how* conceptions about rural areas are related to experiences from specific countries. On the other hand, this heterogeneity makes it possible to capture a wider breadth of variation. The MPs had little experience with rural areas in either Sweden or previous countries. However, conceptions still existed as these are a mixture of one’s own experiences as well as information from elsewhere, such as from teachers, colleagues and media (cf. [18, 19]).

## Conclusions

MP´s holds various conceptions regarding working as a physician in rural and remote areas of Sweden, and regardless of how accurate these conceptions are, they nonetheless influence the MPs’ willingness to work in such areas. Willingness is also affected by, and intertwined with, other aspects such as the MPs previous experiences, age, marital status and family circumstances. Our findings show that rural areas are often considered to have less to offer compared with cities, regarding both professional and private life. The results of this study may provide a basis for further research to explore how common these conceptions are and to what degree MPs’ previous experiences, age and family circumstances are interconnected with them. The findings can be used to develop interventions to address these conceptions in a more positive way, as well as to help reduce the shortage of physicians in rural areas.

## Supporting information

S1 Interview guide (DOCX)
