# Supplementary material for: Migrant physicians’ conceptions of working in rural and remote areas in Sweden: A qualitative study
Source: PLoS One. 2019 Jan 14;14(1):e0210598. doi: 10.1371/journal.pone.0210598 (PMC6331096; doi:10.1371/journal.pone.0210598)
Supplement: S1 Interview guide — (DOCX) [file pone.0210598.s001.docx]

## Interview Guide

The interviews were semi-structured. Therefore, the Interview guide featured a number of themes and some pre-set questions. These questions were used as an introduction to the themes. Follow-up questions were adapted to respondents' answers. Below are the themes and the initial questions for each respective theme presented:

Background

- How old are you?
- Where are you from?
- Where have you grown up?
- Do you live alone or do you live with someone?

Medical career – previous country

- Where did you have your medical education?
- Did you work as a physician in [former country] or elsewhere before you came to Sweden?
  How long did you work as a physician?
  Have you worked as a specialist? Within what specialty?

Where did you work?
For what reasons did you work there?

Work as a physician in cities, smaller communities and in rural areas – former country

- Do you think there are any differences between working as a doctor in cities, in smaller communities and in rural areas in [former countries]?

Work as a physician in cities, smaller communities and in sparsely populated/rural areas – Sweden

- Here in Sweden, do you think there are any differences between working as a physician in a city and in rural areas?

Future career

- What do you want your future career to look like?
- Where is it possible for you to pursue this career?
- What do you think about the possibility of having this career in a rural area in Sweden in comparison to a city?

To Sweden

- When did you come to Sweden?
- Why did you move to Sweden?
- Please tell where in Sweden you have lived and why you lived there.

Experience of work in Sweden

- Have you worked in Sweden before the complementary programme for physicians?
  If yes, please tell within what and as what.

## Intervjuguide

Intervjuerna var semistrukturerade. Intervjuguiden innehöll ett antal teman och frågor. De färdiga frågorna användes för att skapa en ingång till varje tema. Uppföljningsfrågorna anpassades efter respondenternas svar. Nedan presenteras tema och ingångsfrågorna till varje tema:

Bakgrund

- Hur gammal är du?
- Varifrån kommer du?
- Var är du uppvuxen?
- Lever du ensam eller lever du med någon?

Läkarkarriären – tidigare land

- Var utbildade du dig till läkare?
- Hann du arbeta som läkare i [tidigare land] eller någon annanstans innan du kom till Sverige?

Hur länge arbetade du som läkare?
Har du arbetat som specialist? Inom vad?

- Var någonstans arbetade du?

Hur kom det sig att du arbetade just där?

Arbete som läkare i städer, mindre samhällen och på ladsbygd/glesbygd – tidigare land

- Tänker du att det finns skillnader mellan att arbeta som läkare i större städer eller mindre samhällen och på landsbygden/glesbygden i [tidigare land]?

Arbete som läkare i städer, mindre samhällen och på ladsbygd/glesbygd – Sverige

- Här i Sverige, tror du att det finns det några skillnader mellan att arbeta som läkare i en stor stad och på landsbygden?

Framtida karriär

- Hur vill du att din framtida karriär ska se ut?
- Var är det möjligt för dig att göra denna karriär?
- Hur tänker du kring möjligheterna för dig att få denna karriär på landet eller glesbygden här i Sverige i jämförelse med i en stad?

Till Sverige

- När kom du till Sverige?
- Hur kommer det sig att du flyttade till Sverige?
- Berätta var i Sverige du har bott någonstans och varför du bott där.

Erfarenhete av arbete i Sverige

- Har du arbetat inom vården i Sverige innan den kompletterande utbildningen?

Om ja, berätta inom vad och som vad.
